# Quantifying seasonal and diel variation in Anopheline and Culex human biting rates in Southern Ecuador

**DOI:** 10.1186/s12936-017-2121-4

**Published:** 2017-11-22

**Authors:** Sadie J. Ryan, Catherine A. Lippi, Philipp H. Boersch-Supan, Naveed Heydari, Mercy Silva, Jefferson Adrian, Leonardo F. Noblecilla, Efraín B. Ayala, Mayling D. Encalada, David A. Larsen, Jesse T. Krisher, Lyndsay Krisher, Lauren Fregosi, Anna M. Stewart-Ibarra

**Affiliations:** 1 Emerging Pathogens Institute, University of Florida, Gainesville, FL USA; 2 Department of Geography, University of Florida, Gainesville, FL USA; 3 Center for Global Health and Translational Science and Department of Medicine, State University of New York Upstate Medical University, Syracuse, NY USA; 4 College of Agriculture, Engineering, and Science, University of KwaZulu-Natal, Durban, South Africa; 5 Department of Integrative Biology, University of South Florida, Tampa, FL USA; 6 Laboratorio Clínico Hospital Teófilo Dávila, Ministerio de Salud Pública, Machala, Ecuador; 7 Lab. Entomologia CZ7, Ministerio de Salud Pública, Machala, Ecuador; 8 Facultad de Medicina, Universidad Técnica de Machala, Machala, Ecuador; 9 Dirección Nacional de Vigilancia Epidemiológica, Ministerio de Salud Pública, Av. República de El Salvador 36-64 y Suecia, 170515 Quito, Ecuador; 10 Department of Public Health, Food Studies, and Nutrition, Syracuse University, Syracuse, NY USA; 11 Division of Nutritional Sciences, Cornell University, Ithaca, NY USA; 12 Center for Health, Work & Environment, Department of Environmental and Occupational Health, Colorado School of Public Health, University of Colorado Denver, Aurora, CO USA; 13 Colorado Consortium on Climate Change and Human Health, University of Colorado Denver, Aurora, CO USA

**Keywords:** *Anopheles albimanus*, *Anopheles punctimacula*, Bite rate, Ecuador, Malaria, Culex

## Abstract

**Background:**

Quantifying mosquito biting rates for specific locations enables estimation of mosquito-borne disease risk, and can inform intervention efforts. Measuring biting itself is fraught with ethical concerns, so the landing rate of mosquitoes on humans is often used as a proxy measure. Southern coastal Ecuador was historically endemic for malaria (*Plasmodium falciparum* and *Plasmodium vivax*), although successful control efforts in the 2000s eliminated autochthonous transmission (since 2011). This study presents an analysis of data collected during the elimination period.

**Methods:**

Human landing catch (HLC) data for three mosquito taxa: two malaria vectors, *Anopheles albimanus* and *Anopheles punctimacula*, and grouped *Culex* spp. were examined for this study. These data were collected by the National Vector Control Service of the Ministry of Health over a 5-year time span (2007–2012) in five cities in southern coastal Ecuador, at multiple households, in all months of the year, during dusk–dawn (18:00–6:00) hours, often at both indoor and outdoor locations. Hurdle models were used to determine if biting activity was fundamentally different for the three taxa, and to identify spatial and temporal factors influencing bite rate. Due to the many different approaches to studying and quantifying bite rates in the literature, a glossary of terms was created, to facilitate comparative studies in the future.

**Results:**

Biting trends varied significantly with species and time. All taxa exhibited exophagic feeding behavior, and outdoor locations increased both the odds and incidence of bites across taxa. *Anopheles albimanus* was most frequently observed biting, with an average of 4.7 bites/h. The highest and lowest respective months for significant biting activity were March and July for *An. albimanus,* July and August for *An. punctimacula*, and February and July for *Culex* spp.

**Conclusions:**

Fine-scale differences in endophagy and exophagy, and temporal differences among months and hours exist in biting patterns among mosquito taxa in southern coastal Ecuador. This analysis provides detailed information for targeting vector control activities, and household level vector prevention strategies. These data were collected as part of routine vector surveillance conducted by the Ministry of Health, and such data have not been collected since. Reinstating such surveillance measures would provide important information to aid in preventing malaria re-emergence.

**Electronic supplementary material:**

The online version of this article (10.1186/s12936-017-2121-4) contains supplementary material, which is available to authorized users.

## Background

Despite major efforts to control and eliminate vector-borne diseases through vector control, mosquito-borne diseases such as malaria, dengue, yellow fever, and now chikungunya and zika virus remain a major threat to people’s livelihoods in the Americas. An estimated 108 million people per year are at risk for malaria infections in the Americas, pointing to a need to maintain elimination status in areas that have successfully eliminated local infections, and to prevent reestablishment [1]. In Latin America there is high endemic diversity in both vectors and pathogens, including three species of malaria-causing parasites, *Plasmodium vivax*, *Plasmodium falciparum*, and *Plasmodium malariae* [1–4]. To monitor and measure the potential for mosquito-borne transmission, it is important to assess the risk or rate of infectious bites on humans. There are many challenges associated with the direct surveillance of pathogens, such as *Plasmodium*, in mosquito populations, thus vector-borne diseases are often monitored in terms of human case data [5–7]. The reliance on human cases to monitor vector-borne disease outbreaks is subject to many forms of reporting bias, and these biases may be further exacerbated in Ecuador, where disparities in clinical access may contribute to underreporting of cases, as is seen with dengue [8–10]. Even when clinical access is more widely available, as in urban areas, much of the public health data reported by Ecuador’s Ministry of Health relies on suspected clinical cases rather than laboratory confirmation [11]. Furthermore, human case data does not provide information in sufficient time to target vector control to mosquito activity. Although malaria surveillance and diagnostics in Ecuador are much stronger relative to those of other mosquito-borne diseases, detection of asymptomatic malaria and cases in remission remain a challenge to surveillance and disease elimination [12, 13].

Measuring force of infection, or transmission risk of mosquito-borne diseases through models of vital rates [14–17], require knowledge of many components of the transmission cycle, including biting rates. The entomological inoculation rate (EIR) is commonly used as a means of describing potential risk of infection from vector-borne diseases; this is the rate of infectious bites per person per day, usually estimated, or derived from biting rates and a measure of vector infection prevalence. EIR is considered a more direct measure of infection intensity than human incidence or other traditional epidemiological measures [18, 19]. However, in low-transmission situations, estimating sporozoite rates is stymied by large statistical error range, and thus biting rate is a better means of estimating transmission. Clearly, measuring the rate of infection in vectors can be logistically complex, but capturing an estimate of biting rate, perhaps less so. Thus, a simplified attempt to quantify potential disease transmission is the development of human bite rate (HBR) and landing rate (LR) indices, generally described as the number of mosquitoes of a species respectively exhibiting feeding or resting behaviour on a human recorded for a given location and time period [20–22]. Although used for estimating the number of female mosquitoes that are attempting to take blood meals in field or laboratory conditions, there is a great deal of variability in the literature with regards to the definitions and field protocols associated with these metrics.

A glossary of biting rate terms encountered in the literature was developed, to facilitate communication of definitions, as a means to both measure and interpret study findings for comparison (Table 1). In general, the protocol for HBR and LR studies involves an initial survey for potential sites, a species inventory to establish vector presence, training field entomology technicians in identification of species and behaviours, and establishing spatial points and temporal intervals for data collection [23]. Like raw mosquito density, HBR and LR do not directly measure infections, but these indices are often cited as a proxy for species presence, density of blood-seeking females, and the capacity for disease transmission [23, 24]. Potential issues with HBR include reliance on visual identification of mosquito species, inter-observer agreement, and exposure of workers to pathogens [25–28]. Human landing catch (HLC), wherein mosquitoes counted in the landing rate survey are captured and later examined in the lab, can overcome most of these obstacles, but at the cost of additional field and laboratory resources [22]. Depending on study design and data collection protocol, bite rate indices have the potential to provide a wealth of information regarding vector behaviour at very fine spatial and temporal scales in a manner that is both relatively cost-effective and efficient.

**Table 1:** Glossary of terms related to mosquito biting activity used in the literature

Source	Term	Definition	Citations
Field collection	Human biting rate	The number of actively biting or blood fed female mosquitoes divided by the number of study participants per night, sometimes estimated from human landing catch	[21, 22, 46, 53]
	Human biting density	Sum of female mosquitoes caught in a sampling period divided by the total number of houses sample	[53]
	Human landing rate	Number of mosquito bites per night estimated from number of female mosquitoes landing on subjects	[29]
	Biting rate/biting activity/bite trends	Sum of female mosquitoes landing on volunteers averaged for different portions of survey period	[23, 46]
	Human landing catch	Human bait used to capture female mosquitoes as they attempt to feed, usually with an aspirator	[30–33]
	Human landing collection	Human bait used to capture female mosquitoes as they attempt to feed, usually with an aspirator	[34, 35]
	Human landing count	Human bait used to capture female mosquitoes as they attempt to feed, usually with an aspirator	[36]
	Human biting catch	Female mosquitoes captured in the act of biting	[37]
	Man-landing catch	Human subjects act as both baits and traps for female mosquitoes	[38]
Laboratory collection	Mosquito biting rate	The number of female mosquitoes that attempted feeding on a human study participant during an observation period under laboratory or experimental conditions	[39, 40]
Derived model parameters	Biting rate	Parameter used in estimating malaria *Ro* in the Ross-MacDonald model	[24, 41]
	Entomological inoculation rate Human biting rate	Malaria transmission estimated from human bite rate Parameter estimated from human landing catch data collected in the field	[21, 42, 43]

**Fig. 1:**
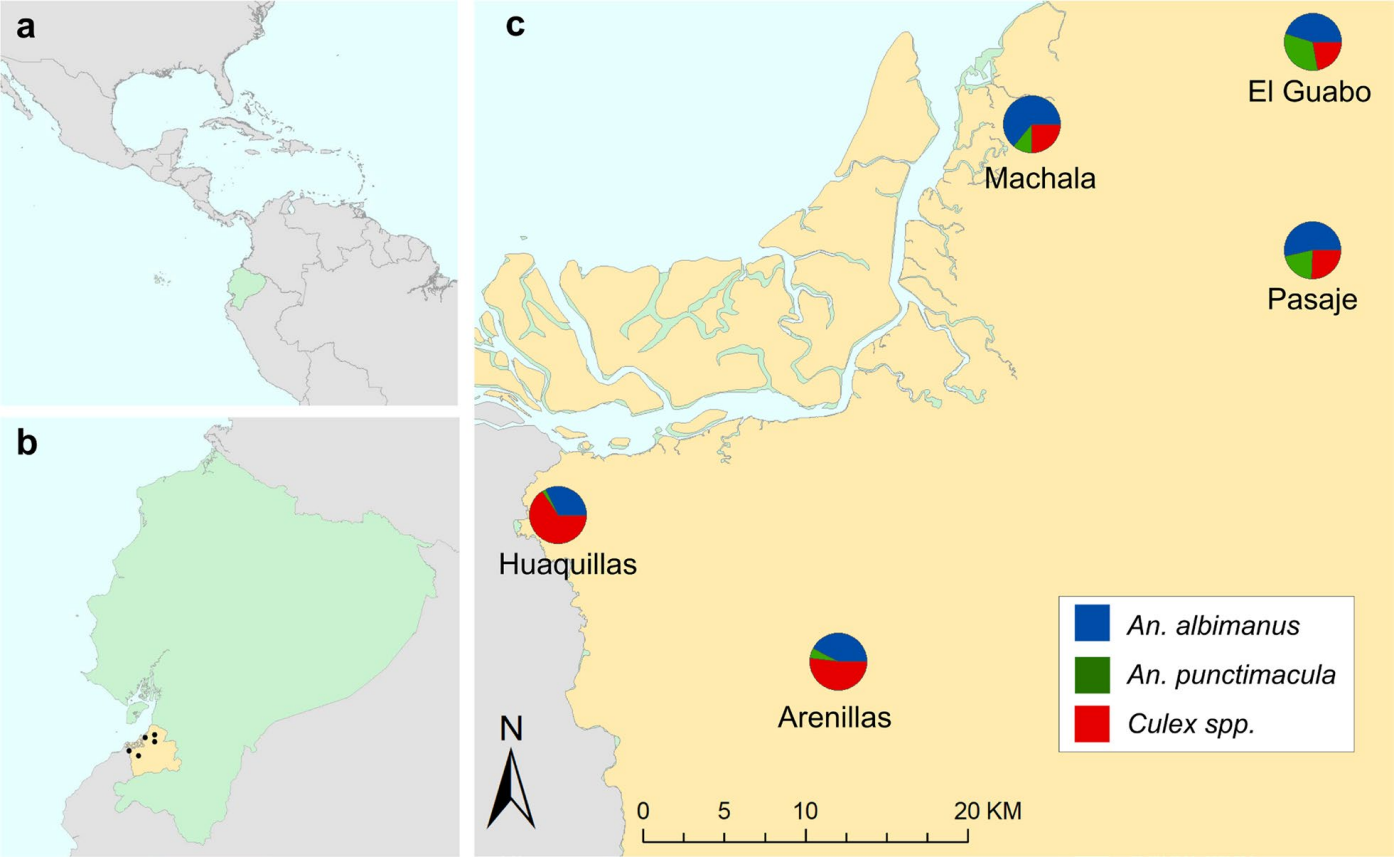
Data on mosquito biting rates were collected in five cities located in Ecuador’s (**a**) southern coastal El Oro province (**b**). Although the proportion of bites recorded relative to sampling effort for *Anopheles albimanus*, *An. punctimacula*, and *Culex* spp. varied between cities, all three taxa of interest were detected across study sites (**c**)

Ecuador’s southern El Oro province (Fig. 1) has been free of locally acquired malaria infections since 2011, although the mosquito species capable of vectoring *P. vivax* and *P. falciparum* malaria are still prevalent in the area [13]. Disease surveillance and control programmes in developing countries typically suffer from limited resources in the face of high disease burden, however the Ecuadorian government has devoted a great deal of funding and logistic support to their Ministry of Health specifically for the detection and control of malaria following a resurgence of the disease in the late 1990s, which has been previously described in detail [13]. Nevertheless, with recent outbreaks of malaria occurring in other Ecuadorian provinces and neighbouring countries, the potential for re-emergence of malaria in El Oro creates a need to estimate the potential for malaria transmission as part of a surveillance system, and the behaviour of blood-seeking female mosquitoes recorded via HLC can enhance the understanding of outbreak and exposure risks by illuminating relevant aspects of vector biology, such as seasonal activity trends by species, peak biting activity by species, detailed shifts in species composition, and host-seeking behaviour and the propensity for endophagy (indoor feeding) [44–48]. This is information that can be directly incorporated into mosquito abatement strategies, surveillance protocols, and public education campaigns.

Previous bite rate studies on *Anopheles* have demonstrated that mosquitoes can shift species composition and peak daily biting activity in response to abatement strategies, information that is crucial to developing and reviewing successful mosquito control efforts [21, 49–51]. In Ecuador, there have been documented instances of epidemiological shifts in human disease patterns with concurrent transitions in species prevalence, and long-term collection of bite rate data at fine scales can capture these shifts [52]. This is an important consideration, as biting rate and peak biting activity are often considered as stable variables for any given species that can be directly reduced through routine interventions [18, 24, 53].

In this study, nightly bite rate data collected in five cities from 2007 to 2012 in southern Ecuador, was examined. These data were collected as part of routine *Anopheline* surveillance by the National Service for the Control of Diseases Transmitted by Arthropod Vectors (SNEM) of the Ministry of Health. The goals of this paper are to (1) test the hypothesis that the bite indices for notable mosquito vectors in southern coastal Ecuador differ significantly across taxa (2) use an exploratory modelling framework to describe seasonal and diel variation in biting activity within each taxon and (3) use fine-scale data to compare exophagic and endophagic feeding behaviours between taxa.

## Methods

### Bite rate data

Human landing catch (HLC) data were collected as a proxy for the biting activity (i.e. bite rate) of two malaria vectors (*Anopheles albimanus* and *Anopheles punctimacula*) and a pooled taxonomic grouping of potential arbovirus vectors (*Culex* spp.) at the household level from 2007 to 2012 in five coastal cities in Ecuador’s El Oro province: Huaquillas, Machala, El Guabo, Arenillas, and Pasaje (Fig. 1). In the first year of study, three primary sites (Huaquillas, Machala, and El Guabo) were surveyed every month to establish baseline data. In subsequent years, each site was surveyed four times annually, twice in the rainy season (January–May) and twice in the dry season. Field technicians were equipped with black stockings that covered the legs from the feet to above the knees and captured mosquitoes landing on the stockings with a mouth aspirator. Hourly collections were made each night (18:00–06:00) at study households, both inside homes and outdoors, allotting 50 min of each hour for aspiration and 10 min for specimen processing. All mosquitoes collected were brought back to the laboratory for counting, sexing, and species identification. Although sampling effort (i.e. number of survey nights) varied between cities [Arenillas (n = 17), El Guabo (n = 27), Huaquillas (n = 38), Machala (n = 33), Pasaje (n = 2)], all three mosquito taxa were detected in all study sites (Fig. 1).

### Statistical analysis

Regression models were used to determine if bite rates were fundamentally different for the three mosquito taxa, and to explore the influence of biting location (i.e. indoors vs. outdoors), season, and time of biting activity (i.e. hour of the night). Due to the size of the data set, limiting the capacity to detect city-level differences data were pooled across the five cities in the study. The bite rate data exhibited more zero observations than accommodated by commonly used error distributions for count data (e.g. Poisson or negative Binomial), an issue frequently encountered when modeling mosquito surveillance datasets, but not always treated in a statistically appropriate manner. Hurdle models were used, which combine a logistic regression model, the so-called hurdle, which describes the probability of being bitten at all, with a count model, which describes the number of bites conditional on being bitten [54]. In addition to wishing to use the appropriate statistics for the zero observations, hurdle models were also used rather than zero-inflated Poisson (ZIP) models, due to the inability to distinguish between “structural” and “sampling” zeroes in these data. In this specific case, this leads to superior interpretability, allowing for direct modelling of the probability of being bitten by a particular species.

Hurdle models were fitted using the package ‘pscl’ in R ver. 3.3.1 (R Core Team, 2016), specifying a negative binomial error distribution and a log link for the count component, and a binomial error distribution and a logit link for the hurdle [55]. Variable selection for hurdle models was conducted based on Akaike’s Information Criterion [56]. Confidence intervals for model predictions were obtained using non-parametric bootstrapping with the ‘boot’ package in R [57, 58].

## Results

Biting behaviour for *An. albimanus, An. punctimacula,* and *Culex* spp. differed, both in terms of whether or not bites occurred (i.e. the odds ratio (OR) of being bitten) and the number of bites/h conditional on being bitten (expressed as incidence rate ratios, RR; Table 2). *Anopheles albimanus* was the species most commonly observed biting (Fig. 3). The occurrence of *An. albimanus* bites in a given hour was four times as likely as no bites (OR 4.04, p < 0.001), with an average of 4.7 bites/h (RR 4.74, p < 0.001).

**Table 2:** Species and location effects of a hurdle model of hourly biting rates

	Count model rate ratio	Zero model odds ratio
Intercept (*An. albimanus*)	4.74 (3.05–7.36)***	4.04 (2.39–6.82)***
*Culex* spp.	1.38 (0.79–2.42)	3.31 (1.58–6.92)**
*An. punctimacula*	0.6 (0.31–1.18)	0.65 (0.31–1.36)
Location outdoors	1.55 (1.36–1.75)***	2.32 (2.03–2.64)***
*Culex*: outdoors	0.79 (0.66–0.94)**	0.58 (0.48–0.7)***
*An. puncti*.: outdoors	0.9 (0.71–1.13)	0.8 (0.66–0.97)*

Model coefficients are presented as incidence rate ratios for the count model (which models hourly bites conditional on being bitten), and as odds ratios for the zero model (which models the probability of being bitten). A full table including the species-specific temporally resolved model coefficients is presented in the supplementary materials. Coefficients in this are representative for January at 6 p.m. local time and relative *to An. albimanus* bite rates. Values in parentheses are 95% confidence intervals. Significance levels are * p < 0.05, ** p < 0.01, *** p < 0.001

Being outdoors more than doubled the odds of being bitten by *An. albimanus* (OR 2.32, p < 0.001), and increased the number of bites received when bitten by about 50% (RR 1.55, p < 0.001). For *Culex* spp. the odds of being bitten were lower overall (Fig. 3), albeit higher at the temporal reference levels of the model (i.e. January at 6 p.m.) with an odds ratio of being bitten by *Culex* of 13.27 (p < 0.01) and an average of 6.5 bites when bitten (n.s. compared to *An. albimanus*). Being outdoors increased the odds of being bitten by *Culex* by about a third (OR 1.35, p < 0.01), and number of bites received by about a quarter (RR 1.22, p < 0.01), both to a lower extent than the associated increases for *An. albimanus*.

Bite rates for *An. punctimacula* were the lowest overall (Fig. 3), with a baseline odds ratio of being bitten of 2.62 and 2.94 bites/h, but these base rates did not differ significantly from those for *An. albimanus*. Being outdoors increased the risk of being bitten by *An. albimanus* by about 80% (or 1.86, p < 0.05), and receiving bites by 40% (RR 1.40, n.s. compared to *An. albimanus*).

Months of peak high and low biting activity varied for the three taxa; the highest and lowest respective months for significant biting activity were March and July for *An. albimanus,* July and August for *An. punctimacula*, and February and July for *Culex* spp. (Table 3).

**Table 3:** Predicted average nightly bite rates (bites/hour) and associated 95% confidence intervals

Month	*An. albimanus*		*An. punctimacula*		*Culex* spp.	
	Indoors	Outdoors	Indoors	Outdoors	Indoors	Outdoors
Jan	4.85 (2.57–8.01)	8.06 (4.98–12.52)	3.59 (1.53–6.06)	5.26 (2.51–8.69)	9.28 (5.85–11.99)	11.31 (7.34–14.64)
Feb	1.93 (0.78–3.58)	3.73 (1.84–6.32)	0.68 (0.19–1.27)	1.29 (0.38–2.37)	8.12 (5.28–10.43)	9.8 (6.51–12.63)
Mar	9.73 (5.42–15.88)	15.96 (10.05–24.75)	1.03 (0.34–1.77)	1.69 (0.64–2.85)	2.69 (1.33–3.8)	3.45 (1.8–4.69)
Apr	3.3 (1.66–5.54)	5.6 (3.34–8.78)	0.38 (0.11–0.68)	0.71 (0.21–1.28)	3.89 (2.24–5.01)	4.81 (2.9–6.13)
May	3.13 (1.6–5.21)	5.26 (3.18–8.19)	0.07 (0.03–0.11)	0.13 (0.05–0.18)	0.93 (0.39–1.49)	1.23 (0.55–1.9)
Jun	5.31 (3.18–8.29)	8.31 (5.5–12.48)	0.24 (0.08–0.4)	0.43 (0.14–0.72)	4.4 (2.93–5.54)	5.25 (3.58–6.63)
Jul	2.85 (1.49–4.67)	4.71 (2.9–7.24)	1.69 (0.78–2.67)	2.39 (1.24–3.72)	0.77 (0.35–1.18)	0.99 (0.47–1.46)
Aug	1.69 (0.65–3.22)	3.38 (1.59–5.88)	0.58 (0.17–1.05)	1.06 (0.33–1.9)	2.47 (1.19–3.53)	3.18 (1.63–4.38)
Sep	7.27 (3.56–12.53)	12.68 (7.41–20.35)	1.87 (0.53–3.52)	3.34 (1.08–6.11)	3.35 (1.6–4.8)	4.35 (2.2–5.99)
Oct	6.59 (3.44–11.02)	11.1 (6.77–17.44)	2.98 (0.71–6.01)	5.88 (1.56–11.54)	1.24 (0.57–1.87)	1.6 (0.78–2.33)
Nov	3.14 (1.38–5.63)	5.77 (3.09–9.51)	1.32 (0.33–2.59)	2.58 (0.71–4.95)	3.32 (1.7–4.57)	4.23 (2.28–5.61)
Dec	1.9 (0.95–3.15)	3.19 (1.9–4.93)	1.06 (0.37–1.79)	1.69 (0.68–2.79)	2.16 (0.83–3.6)	2.99 (1.21–4.8)

## Discussion

Using data collected during a 5 year period across five cities in southern Ecuador, temporal differences in the biting activity and endophagous versus exophagous behavior of mosquito taxa, including two species of known medical significance in Ecuador, were quantified [13, 59, 60]. *Anopheles albimanus*, a noted vector of malaria in Latin America, was the species most frequently observed attempting to bite human subjects, and although the baseline odds of being bitten by this species did not differ significantly from the other malaria vector, *An. punctimacula*, there are still distinct patterns of seasonal and temporal biting activity between the species (Tables 2, 3; Additional file 1). Despite these observed differences, all taxa demonstrated exophagic feeding tendencies—being outside of households increased the risk of exposure to mosquito bites regardless of species (Table 3).

**Fig. 2:**
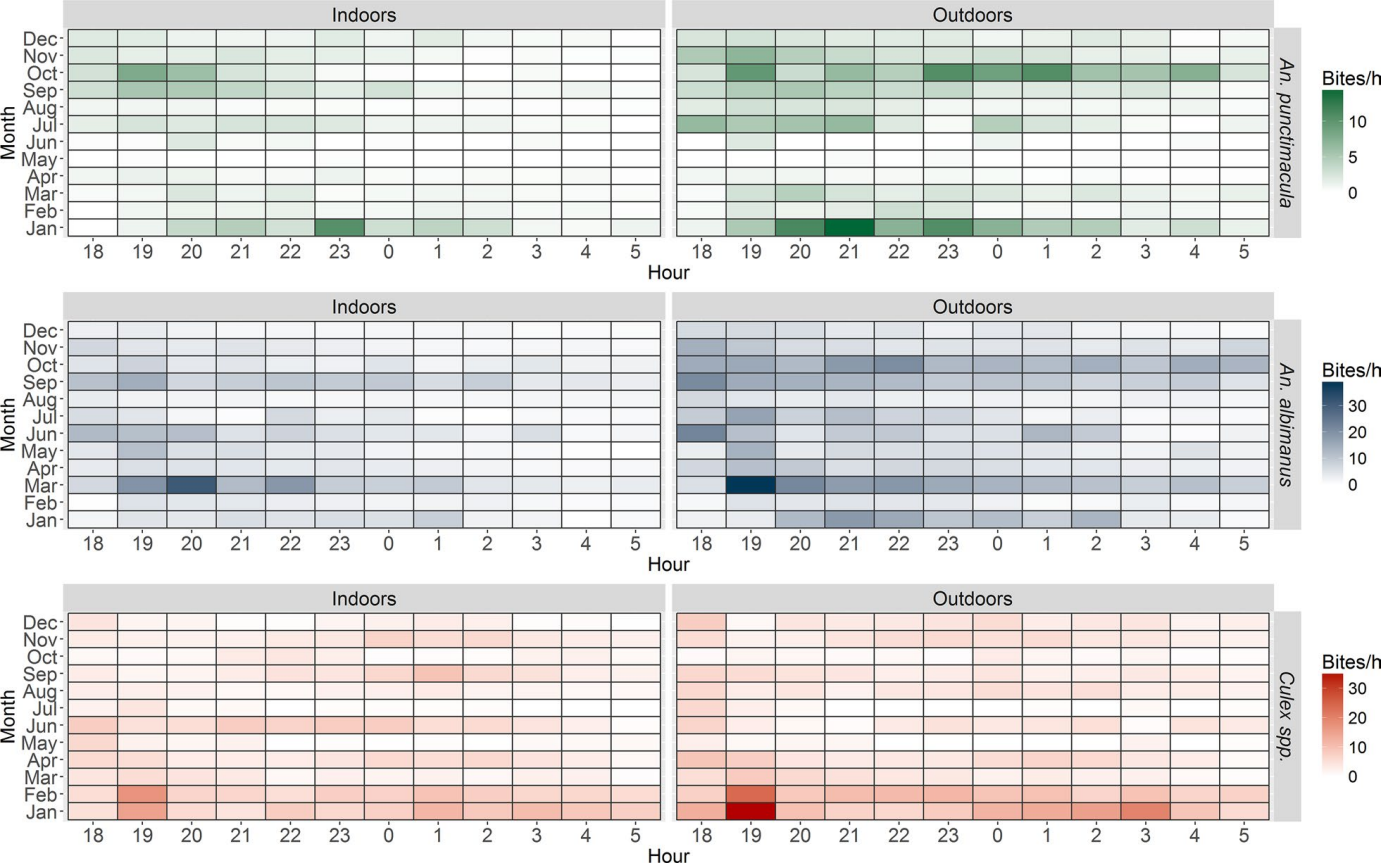
Raw observations of average hourly bite rates by species and location

**Fig. 3:**
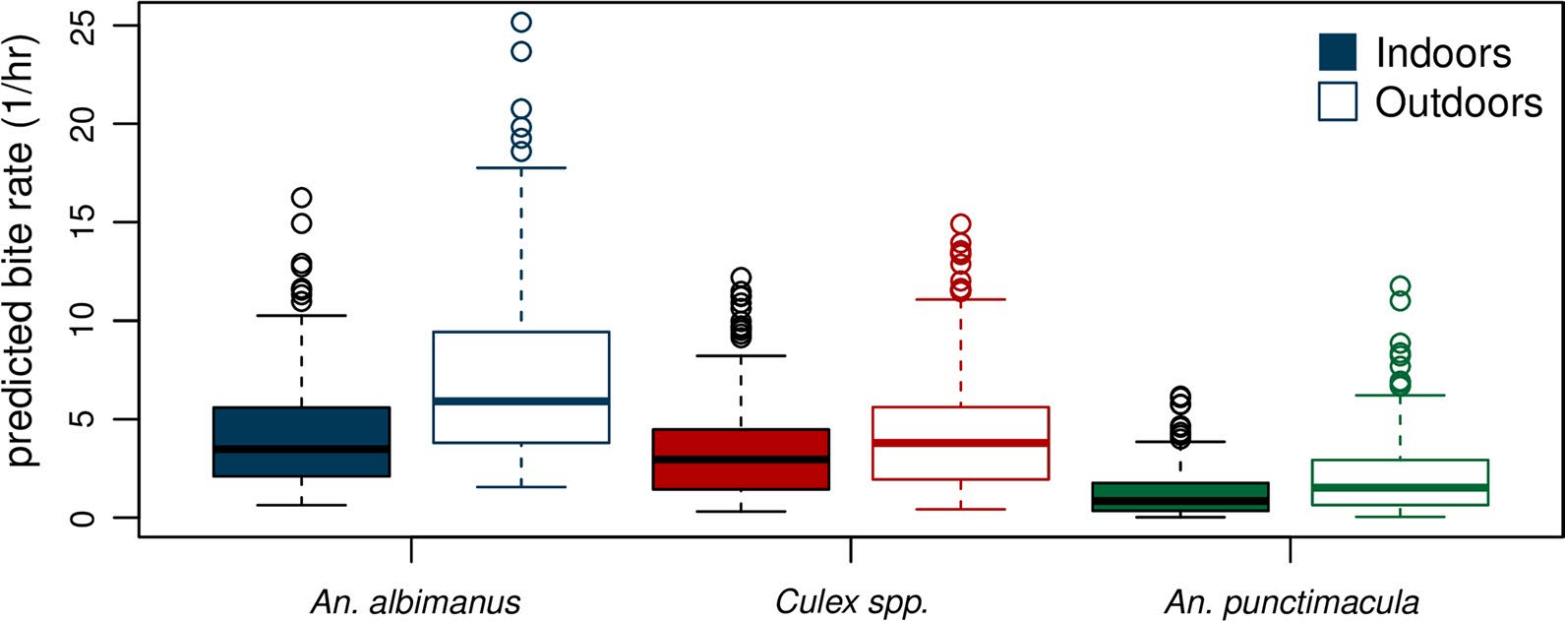
Hourly bite rates by species and location as predicted by the hurdle model across all months and hours of the night

These findings have clear implications for the delivery of mosquito abatement services and the development of public outreach programmes, as risk of exposure to mosquito bites is a demonstrated function of time (e.g. month, hour of activity), location (i.e. indoors vs. outdoors), and species of vector (Figs. 2, 3). The hot rainy season occurs from January to April, and historically, malaria season was around March–July, peaking in May [13]. Given that there was highest biting activity for *An. albimanus* in March, and lowest in July, but highest in July and lowest in August for *An. punctimacula*, the human exposure to these anopheline biting habits suggests a mix of activity level between the two species during the malaria season. For areas such as El Oro province, where malaria has been eliminated, a priori knowledge of exposure risks can be incorporated into a framework of targeted surveillance and control to prevent reemergence or reestablishment of malaria in the region. There is active vector control (household spraying) year round in Ecuador, but mosquito control efforts intensify and focus immediately before and during the rainy season (January–May), when increased water availability provides ample habitat for the aquatic larval stages of mosquitoes. Such interventions are either focused on reducing overall mosquito abundance or targeted on pooled taxonomic groupings (e.g. managing malarial infections by treating the genus *Anopheles* as a single group). Biting activity of the primary malaria vectors extends beyond the focal spray season—particularly *An. punctimacula*, which has peak activity a full 2 months after focal activity is finished. This could potentially allow additional malaria activity later in the season, and increase the role of the vector thought to be less important in Latin America. Incorporating temporal biting trends by species into management plans (i.e. peak months of biting activity) has the potential to increase the effectiveness and efficiency of mosquito control programmes by allowing decision-makers to focus resources at time periods critical to disrupting life cycles of particular vectors, and consequently the diseases they spread.

The dynamics of malaria transmission in Latin American countries are complex, and to fully understand localized disease risks, exposure to vectors and also the vectorial capacity of mosquitoes, must be examined, which can vary with species and environment [61–63]. That said, quantifying taxonomic-specific biting patterns is still a useful endeavour when developing control strategies, as demonstrably competent disease vectors are known to display differential feeding behaviours throughout their geographic ranges. This is the case with *An. albimanus*, which has been observed displaying both anthropophilic and zoophilic feeding preferences depending on location, potentially responsible for spatial variability in the true risk of disease transmission to humans [2, 64–66]. Similarly, patterns of microhabitat use can vary spatially, with the proportion of endophagic versus exophagic mosquitoes depending not only on taxon, but also spatially contextual factors such as environment and housing structures [47, 66]. In these instances, the collection of HLC data can serve as a better indicator of true exposure risk than simply documenting the presence of known competent vectors.

The utility of bite rate indices as a relatively low-cost surveillance tool is well documented [22, 23, 65]. However, the ability to differentiate closely related mosquito species may serve as an additional logistical challenge to the field surveillance of mosquito vectors in Ecuador. Female *An. punctimacula* are morphologically similar to *Anopheles calderoni*, another vector of malaria in Latin America [67]. Despite being a competent vector of *Plasmodium* spp., *An. calderoni* was only recently confirmed in several Latin American countries, including Ecuador, due to the systematic misclassification of the species [67, 68]. The potential for misidentification of these taxa on surveys may obscure true species-level patterns in biting activity. Given the combination of later season biting activity, and potential misidentification, this warrants future work.

The bite count data in this study were collected at a very high temporal (e.g. hourly) and behavioural level (e.g. inside and outside of households) resolutions but were pooled across the five study cities for statistical analysis. This was largely due to the high number of variable combinations (e.g. species by month, species by hour) relative to the number of collection nights and the inherent zero-inflated nature of count data. Ideally, future studies would strive for more spatio-temporally balanced data collection across cities, allowing for more robust exploration of the larger spatial variation (inter-city) in biting trends across the study region. This would involve deploying multiple trained teams, which may be a prohibitive constraint at present. Despite these limitations, human bite rate indices remain a valuable tool in the collection of high-resolution vector ecology data, enabling quantification of risks associated with exposure to mosquito bites in a manner that is cost-effective and simple to implement.

## Conclusions

This is the first time that fine-scale behavioural (endophagy and exophagy) and temporal differences in the biting patterns of mosquito taxa have been reported for El Oro province in southern coastal Ecuador. These findings provide detailed information for targeting vector control and household level prevention strategies. Quantifying hourly and seasonal biting activity, and examining endo- and exophagous behaviours are important to allocating resources and strategies appropriately. The data used to examine human biting trends were collected as part of routine vector surveillance conducted by the Ministry of Health, but such data have not been collected since the end of this dataset. As seen with dengue in the region, even when there is decline in cases, as happened prior to the 1970s, relaxing vector control, and reducing surveillance, can lead to rapid reemergence. Reinstating such surveillance measures will provide important information that will aid in preventing malaria re-emergence.

## Additional file

**Additional file 1: Table S1.** Hurdle model of hourly biting rates. Model coefficients are presented as incidence rate ratios for the count model (which models hourly bites conditional on being bitten using a negative binomial error distribution and log link), and as odds ratios for the zero model (which models the probability of being bitten using binomial errors and a logit link). Values in parentheses are 95% confidence intervals. Significance levels are * p < 0.05, ** p < 0.01, *** p < 0.001.

## Abbreviations

HLC: human landing catch
EIR: entomological inoculation rate
HBR: human biting rate
LR: landing rate
SNEM: National Service for the Control of Diseases Transmitted by Arthropod Vectors (Ecuador)
OR: odds ratio
RR: rate ratio
