# Supplementary material for: Quantifying seasonal and diel variation in Anopheline and Culex human biting rates in Southern Ecuador
Source: Malar J. 2017 Nov 22;16:479. doi: 10.1186/s12936-017-2121-4 (PMC5700746; doi:10.1186/s12936-017-2121-4)
Supplement: Supplementary file 1 — Additional file 1: Table S1. Hurdle model of hourly biting rates. Model coefficients are presented as incidence rate ratios for the count model (which models hourly bites conditional on being bitten using a negative binomial error distribution and log link), and as odds ratios for the zero model (which models the probability of being bitten using binomial errors and a logit link). Values in parentheses are 95% confidence intervals. Significance levels are * p < 0.05, ** p < 0.01, *** p < 0.001. [file 12936_2017_2121_MOESM1_ESM.docx]

**Table S1:** Hurdle model of hourly biting rates. Model coefficients are presented as incidence rate ratios for the count model (which models hourly bites conditional on being bitten using a negative binomial error distribution and log link), and as odds ratios for the zero model (which models the probability of being bitten using binomial errors and a logit link). Values in parentheses are 95% confidence intervals. Significance levels are P < 0.05 *, P < 0.01 **, P < 0.001 ***.

|  | Count model rate ratio | Zero model odds ratio |
| --- | --- | --- |
| (Intercept) | 4.74 (3.05-7.36) *** | 4.04 (2.39-6.82) *** |
| Speciesculex | 1.38 (0.79-2.42) | 3.31 (1.58-6.92) ** |
| Speciespunti | 0.6 (0.31-1.18) | 0.65 (0.31-1.36) |
| Locationoutdoors | 1.55 (1.36-1.75) *** | 2.32 (2.03-2.64) *** |
| MonthFeb | 0.52 (0.34-0.79) ** | 0.31 (0.19-0.52) *** |
| MonthMar | 2.18 (1.4-3.39) *** | 1.4 (0.79-2.49) |
| MonthApr | 0.66 (0.45-0.99) * | 0.74 (0.45-1.22) |
| MonthMay | 0.6 (0.36-1) . | 0.78 (0.42-1.46) |
| MonthJun | 0.96 (0.56-1.65) | 1.73 (0.81-3.68) |
| MonthJul | 0.51 (0.26-1) * | 0.83 (0.37-1.88) |
| MonthAug | 0.5 (0.33-0.75) *** | 0.26 (0.16-0.42) *** |
| MonthSep | 1.82 (1.22-2.73) ** | 0.8 (0.48-1.32) |
| MonthOct | 1.49 (0.87-2.55) | 1 (0.51-1.97) |
| MonthNov | 0.79 (0.53-1.17) | 0.47 (0.29-0.76) ** |
| MonthDec | 0.32 (0.21-0.48) *** | 0.62 (0.37-1.03) . |
| Hour_factor19 | 1.05 (0.8-1.37) | 1.42 (1-2.02) * |
| Hour_factor20 | 0.8 (0.61-1.06) | 1.17 (0.83-1.64) |
| Hour_factor21 | 0.77 (0.58-1.01) . | 0.84 (0.61-1.18) |
| Hour_factor22 | 0.7 (0.53-0.93) * | 0.91 (0.65-1.28) |
| Hour_factor23 | 0.66 (0.5-0.89) ** | 0.59 (0.43-0.82) ** |
| Hour_factor0 | 0.6 (0.45-0.8) *** | 0.6 (0.44-0.83) ** |
| Hour_factor1 | 0.59 (0.44-0.8) *** | 0.41 (0.3-0.57) *** |
| Hour_factor2 | 0.49 (0.36-0.66) *** | 0.39 (0.28-0.53) *** |
| Hour_factor3 | 0.45 (0.33-0.61) *** | 0.36 (0.26-0.49) *** |
| Hour_factor4 | 0.44 (0.32-0.6) *** | 0.27 (0.2-0.37) *** |
| Hour_factor5 | 0.48 (0.35-0.66) *** | 0.23 (0.16-0.31) *** |
| Speciesculex:Locationoutdoors | 0.79 (0.66-0.94) ** | 0.58 (0.48-0.7) *** |
| Speciespunti:Locationoutdoors | 0.9 (0.71-1.13) | 0.8 (0.66-0.97) * |
| Speciesculex:MonthFeb | 1.58 (0.92-2.73) . | 3.89 (1.87-8.12) *** |
| Speciespunti:MonthFeb | 0.88 (0.45-1.71) | 0.37 (0.18-0.76) ** |
| Speciesculex:MonthMar | 0.15 (0.08-0.27) *** | 0.16 (0.08-0.35) *** |
| Speciespunti:MonthMar | 0.15 (0.08-0.3) *** | 0.2 (0.09-0.44) *** |
| Speciesculex:MonthApr | 0.58 (0.35-0.97) * | 0.65 (0.33-1.29) |
| Speciespunti:MonthApr | 0.35 (0.18-0.65) *** | 0.12 (0.06-0.24) *** |
| Speciesculex:MonthMay | 0.22 (0.08-0.58) ** | 0.11 (0.04-0.3) *** |
| Speciespunti:MonthMay | 0 (0-Inf) | 0.04 (0.01-0.13) *** |
| Speciesculex:MonthJun | 0.38 (0.19-0.79) ** | 0.65 (0.22-1.94) |
| Speciespunti:MonthJun | 0.09 (0.03-0.29) *** | 0.05 (0.02-0.13) *** |
| Speciesculex:MonthJul | 0.14 (0.05-0.41) *** | 0.13 (0.04-0.39) *** |
| Speciespunti:MonthJul | 0.64 (0.24-1.66) | 1.06 (0.34-3.31) |
| Speciesculex:MonthAug | 0.62 (0.37-1.04) . | 0.81 (0.41-1.6) |
| Speciespunti:MonthAug | 0.63 (0.34-1.16) | 0.48 (0.24-0.97) * |
| Speciesculex:MonthSep | 0.26 (0.15-0.43) *** | 0.26 (0.13-0.53) *** |
| Speciespunti:MonthSep | 0.57 (0.31-1.04) . | 0.28 (0.14-0.57) *** |
| Speciesculex:MonthOct | 0.1 (0.04-0.21) *** | 0.13 (0.05-0.33) *** |
| Speciespunti:MonthOct | 2.01 (0.79-5.1) | 0.13 (0.05-0.34) *** |
| Speciesculex:MonthNov | 0.5 (0.3-0.83) ** | 0.6 (0.3-1.17) |
| Speciespunti:MonthNov | 1.47 (0.81-2.68) | 0.25 (0.13-0.51) *** |
| Speciesculex:MonthDec | 1.67 (0.92-3.02) . | 0.13 (0.06-0.26) *** |
| Speciespunti:MonthDec | 0.92 (0.49-1.72) | 0.53 (0.26-1.1) . |
| Speciesculex:Hour_factor19 | 0.99 (0.67-1.48) | 0.49 (0.29-0.82) ** |
| Speciespunti:Hour_factor19 | 1.28 (0.79-2.08) | 0.94 (0.59-1.51) |
| Speciesculex:Hour_factor20 | 0.8 (0.53-1.19) | 0.49 (0.29-0.8) ** |
| Speciespunti:Hour_factor20 | 1.51 (0.93-2.46) . | 1.2 (0.75-1.91) |
| Speciesculex:Hour_factor21 | 0.94 (0.62-1.43) | 0.45 (0.27-0.73) ** |
| Speciespunti:Hour_factor21 | 1.24 (0.75-2.03) | 1.48 (0.94-2.35) . |
| Speciesculex:Hour_factor22 | 1.05 (0.7-1.58) | 0.49 (0.3-0.8) ** |
| Speciespunti:Hour_factor22 | 1.1 (0.67-1.8) | 1.33 (0.84-2.11) |
| Speciesculex:Hour_factor23 | 1.27 (0.84-1.92) | 0.86 (0.53-1.4) |
| Speciespunti:Hour_factor23 | 1.34 (0.8-2.24) | 1.65 (1.05-2.61) * |
| Speciesculex:Hour_factor0 | 1.86 (1.23-2.82) ** | 0.85 (0.52-1.38) |
| Speciespunti:Hour_factor0 | 0.89 (0.53-1.48) | 1.54 (0.98-2.43) . |
| Speciesculex:Hour_factor1 | 2.09 (1.37-3.2) *** | 1.03 (0.63-1.66) |
| Speciespunti:Hour_factor1 | 0.99 (0.58-1.7) | 1.76 (1.11-2.79) * |
| Speciesculex:Hour_factor2 | 2.24 (1.46-3.44) *** | 0.98 (0.61-1.59) |
| Speciespunti:Hour_factor2 | 0.98 (0.57-1.7) | 1.8 (1.14-2.85) * |
| Speciesculex:Hour_factor3 | 2.12 (1.37-3.27) *** | 0.87 (0.54-1.41) |
| Speciespunti:Hour_factor3 | 0.88 (0.5-1.54) | 1.81 (1.14-2.88) * |
| Speciesculex:Hour_factor4 | 1.71 (1.09-2.68) * | 0.84 (0.52-1.35) |
| Speciespunti:Hour_factor4 | 1.17 (0.63-2.15) | 1.47 (0.9-2.37) |
| Speciesculex:Hour_factor5 | 1.2 (0.75-1.92) | 0.72 (0.44-1.16) |
| Speciespunti:Hour_factor5 | 0.81 (0.41-1.59) | 1.13 (0.68-1.88) |
